# Increasing uptake of influenza vaccine by pregnant women post H1N1 pandemic: a longitudinal study in Melbourne, Australia, 2010 to 2014

**DOI:** 10.1186/s12884-015-0486-3

**Published:** 2015-03-05

**Authors:** Elizabeth Anne McCarthy, Wendy Elizabeth Pollock, Lauren Tapper, Maree Sommerville, Susan McDonald

**Affiliations:** Department of Obstetrics and Gynaecology, University of Melbourne, Level 3, Mercy Hospital for Women, 163 Studley Road, Heidelberg, Victoria 3084 Australia; La Trobe University, Midwifery and Neonatal Nursing Professorial Unit, Level 2, Mercy Hospital for Women, 163 Studley Road, Heidelberg, Victoria 3084 Australia; Obstetrics Registrar, Mercy Hospital for Women, 163 Studley Road, Heidelberg, Victoria 3084 Australia; Infection Control Co-ordinator, Level 5, Mercy Hospital for Women, 163 Studley Road, Heidelberg, Victoria 3084 Australia

**Keywords:** Pregnancy complications, Infectious/prevention & control, Influenza vaccines/immunology, Trivalent

## Abstract

**Background:**

A Melbourne (Australia) university affiliated, tertiary obstetric hospital provides lay and professional education about influenza vaccine in pregnancy annually each March, early in the local influenza season. Responding to a 2011 survey of new mothers' opinions, the hospital made influenza vaccine freely available in antenatal clinics from 2012. We wished to determine influenza vaccination uptake during pregnancy with these strategies 5 years after 2009 H1N1.

**Methods:**

Face to face interviews based on US Center for Disease Control and Prevention Pregnancy Risk Assessment Monitoring System with new mothers in postnatal wards each July, 2010 to 2014. We calculated recalled influenza vaccine uptake each year and assessed trends with chi square tests, and logistic regression.

**Results:**

We recorded 1086 interviews. Influenza vaccination during pregnancy increased by 6% per year (95% confidence interval 4 to 8%): from 29.6% in 2010 to 51.3% in 2014 (p < 0.001). Lack of discussion from maternity caregivers was a persistent reason for non-vaccination, recalled by 1 in 2 non-vaccinated women. Survey respondents preferred face to face consultations with doctors and midwives, internet and text messaging as information sources about influenza vaccination. Survey responses indicate messages about vaccine safety in pregnancy and infant benefits are increasingly being heeded. However, there was progressively lower awareness of maternal benefits of influenza vaccination, especially for women with risk factors for severe disease.

**Conclusions:**

We observed improving influenza vaccination during pregnancy. There is potential to integrate technology such as text message or internet with antenatal consultations to increase vaccination coverage further.

**Electronic supplementary material:**

The online version of this article (doi:10.1186/s12884-015-0486-3) contains supplementary material, which is available to authorized users.

## Background

Influenza illness threatens lives of pregnant women, new mothers [1] and babies [2]. Influenza vaccination prevents maternal and infant febrile respiratory illness [3] and reduces preterm birth and fetal growth restriction [4]. The World Health Organization prioritises pregnant women for influenza vaccination [5]. Influenza vaccine uptake has been suboptimal in pregnant women [6]. Women and/or maternity caregivers may underestimate illness severity, overestimate vaccine side effects and face logistic obstacles to vaccination during pregnancy [6]. We, like others, promote influenza vaccination during pregnancy with hospital based professional and lay education and deliver seasonal vaccines to consenting staff, inpatients and outpatients at no cost to the person being vaccinated [6]. In the five years since the 2009 H1N1 pandemic wrought serious consequences for pregnant women and their infants, maternity care providers’ awareness of influenza illness and vaccine has increased, but the level of awareness before 2009 may have been close to zero [7]. Having observed an increase in vaccine uptake among pregnant women soon after the 2009 H1N1 pandemic [8], we wished to examine longer term trends in vaccine uptake with current vaccine promotion procedures. Our objective was to review enabling and obstructing factors in promoting influenza vaccination during pregnancy to inform future lay and professional educational efforts.

## Methods

### Setting

The setting was a university affiliated women’s hospital in Melbourne (38° S latitude) with neonatal intensive care facilities and approximately 6,000 births per year, located adjacent to a 400 bed general hospital with a 29 bed adult intensive care unit. The hospital serves local women as well as being one of three hospitals with neonatal intensive care serving about 70,000 births per annum in the state of Victoria, Australia.

### Vaccine policies, procedures and promotion

National public health policies promoted influenza vaccination at all stages of pregnancy during the survey period, reinforced by a November 2011 Royal Australian and New Zealand College of Obstetricians and Gynaecologists (RANZCOG) statement [9]. General practitioners, workplaces and local health authorities are the main providers of seasonal influenza vaccination in Victoria. The study hospital increased professional education, patient information brochures in antenatal clinics and ward areas after the 2010 survey [8] with more readily accessible vaccine supplies for patients from 2012.

### Survey method and instrument

Mercy Health Human Research Ethics Committee approved this audit project and all participants gave verbal consent to anonymous data collection. As previously described [8], we administered a structured interview based on the US Center for Disease Control and Prevention Pregnancy Risk Assessment Monitoring System (PRAMS) Phase 6 (2009) Topic Reference Questionnaire [10] to postnatal inpatient women. Eligible women had given birth to live infants on any day of 2 consecutive weeks each July.

Academic midwifery and obstetric staff interviewed participants 2010 to 2012 and funded research assistants administered survey interviews in 2013 and 2014. We collected non-identifiable data about maternal age, country of birth, gestational age at childbirth, and model of antenatal care as well as whether or not participant women chose influenza vaccination, their reasons for their choice and whether they recall vaccination during pregnancy. From 2012, we also asked for women’s views about text messaging and other information sources about influenza vaccination. A copy of the questionnaire is available as a Additional file 1.

### Statistical methods

Staff availability for quality assurance determined sample size in 2010 and 2011. Observing an increase in influenza vaccine coverage from 30 to 40% between 2010 and 2011 [8], we aimed to duplicate the audit duration each year. Anticipating about 200 births in 14 consecutive days, the sample size is powered to demonstrate an increase in coverage from 30% by 6 % or more with 80% power (1-beta) and 2-sided alpha of 5% [11]. Data were entered and analysed in SPSS (v 21). We used chi-square to test the hypothesis that proportions changed over time and binary logistic regression to estimate the effect of year of audit on recalled vaccine uptake. Because multiple secondary trends were sought concerning women’s reasons for or against vaccination, we focused, conservatively on very low p values (less than 0.01).

## Results

Data were available for 1086 women who participated in the study over five years. *Response rate*s were high: more than 91% of women completed surveys in four of five audit years (2010 (199/212 = 93.9%), 2011 (240/251 = 95.6%), 2013 (253/260 = 97.3%) and 2014 (191/208 = 91.8%). A clerical oversight left only 203 records of 259 postnatal women (79.9%) in 2012 available for analysis. Reasons for non-participation were collected for 2010, 2011 and 2014 (41 of 671 women). Five women (0.8%) suffered perinatal death and were not invited to participate, 23 (3.4%) were discharged home before the interview could be completed, 7 (1.0%) lacked an appropriate interpreter and 6 (0.9%) declined participation.

*Demographic and obstetric* characteristics of respondents were similar to our previous report [8]. Sixty-two per cent of women (676/1086) were Australian born, 9 women (0.8%) identified as Indigenous and 18 (1.7%) completed the survey with interpreter assistance. Thirteen teenaged mothers participated (1.2%) and more than 1 in 4 participants (296 or 27.3%) were aged 35 or older. Most participants used publically funded, hospital-based antenatal care (72.0%). Other women attended general practitioners for shared antenatal care (17.1%), private obstetricians (10.5%) or other services (0.4%). No trends were identified in maternal age structure or proportions of women who were overseas-born, identified as indigenous or used private obstetric care: see Additional file 2. The majority of births (89%) were at gestation 37 to 41 completed weeks, 8.9% were at 20 to 36 + 6 weeks, 0.8% of births at 42 or more weeks and gestational age was missing information for 1% of respondents.

*Influenza vaccination uptake* showed a statistically significant upward gradient from 30% in 2010 to over 50% in 2014 (chi-square for trend p < 0.01. See Table 1). Overall, 461 of 1086 women (42.3%) recalled vaccination during pregnancy. Binary logistic regression modelling showed that the odds of vaccination uptake could be described by a constant and increasing rate according to year of audit (Y) as follows: Log Odds (recalled vaccination) = −0.804 + 0.248(Y), Exp (Y) = 1.28 (95% confidence interval (CI) 1.171 to 1.402) where Y represents the number of years after 2010. The model gives a gradient of 6% increase in uptake per year (95% CI 4% to 8%).

**Table 1:** **Influenza vaccination coverage during pregnancy as judged by recall of women soon after childbirth**

**Survey year**	**Women recalling influenza vaccination during pregnancy (N)**	**Women stating they were not influenza vaccinated during pregnancy (N)**	**Total completing survey (N)**	**Recalled vaccine uptake (%)**
2010	59	140	199	29.6%
2011	95	145	240	39.6%
2012	72	131	203	35.5%
2013	137	116	253	54.2%
2014	98	93	191	51.3%
All years	461	625	1086	42.2%

Pearson Chi-Square = 39.4, df = 4, p < 0.001.

Four hundred and thirteen of 635 women (65.0%) who recalled vaccination being discussed or recommended by a health care worker were subsequently vaccinated compared with 48 of 491 women (10.7%) who did not recall a discussion or recommendation (Pearson chi-square p < 0.001).

Table 2 lists reasons given by women who chose to be vaccinated against influenza during pregnancy. The top ranking reason across the 5 year period was the desire to protect the infant, increasing from 66.7% in 2010 to 89.2% of influenza vaccinated women in 2014 (Pearson Chi-Square p = 0.001).

**Table 2:** **Reasons women gave for electing FOR influenza immunisation in pregnancy** ^**a**^

	**Respondents recalling influenza vaccination during pregnancy**	**I normal haveflu vaccine**		**To prevent maternal illness**		**Chronic medical conditions**		**Midwife recommended vaccination**		**GP recommended vaccination**		**Obstetrician recommended vaccination**		**To protect the baby**	
**Year**	**N**	**n**	**n/N (%)**	**n**	**n/N (%)**	**n**	**n/N (%)**	**n**	**n/N (%)**	**N**	**n/N (%)**	**N**	**n/N (%)**	**n**	**n/N (%)**
2010	60	23	38.3%	39	65.0%	7	11.7%	9	15.0%	39	65.0%	14	23.3%	40	66.7%
2011	94	27	28.7%	50	53.2%	11	11.7%	41	43.6%	39	41.9%	38	41.3%	61	65.6%
2012	72	33	45.8%	37	51.4%	3	4.2%	15	20.8%	33	45.8%	19	26.4%	52	72.2%
2013	139	42	30.2%	28	20.1%	2	1.4%	22	15.8%	58	41.7%	30	21.6%	92	66.2%
2014	93	37	39.8%	52	55.3%	4	4.4%	31	33.3%	34	37.0%	27	28.7%	83	89.2%
	Chi-square, (df)	7.9, (4)		52.2, (4)		14.9, (4)		30.2, (4)		13. 1, (4)		11.7, (4)		18.7, (4)	
	p	0.095		<0.001*		0.005*^b^		<0.001*		0.011		0.020		0.001*	

Results are based on nonempty rows and columns in each innermost sub-table.

*The Chi-square statistic is significant at the 0.01 level.

^a^Women could nominate more than one reason.

^b^More than 20% of cells in this sub-table have expected cell counts less than 5. Chi-square results may be invalid.

The wish to prevent maternal illness was less commonly reported after 2010. Sixty-five per cent cited this reason in 2010; lower proportions (20% to 55%) reported this in subsequent years (p < 0.001). Risk factors for severe maternal influenza disease are common and include obesity, diabetes, smoking and asthma, but were infrequently reported as reasons for immunisation. The proportion of women reporting having a chronic medical condition as a reason for influenza vaccination during pregnancy fell from 11.7% in 2010 to less than 5% in subsequent years (p = 0.005).

Substantial proportions of women reported general practitioner recommendation (44.5%), regular annual immunisation (35.4%) or obstetrician recommendation (28.0%) with no discernible trends over 5 years. Proportions of influenza vaccinated women recalling midwifery recommendations varied widely between audit years but remained higher than the 2010 level (15.0%) in subsequent years (p < 0.001). More than 1 in 4 (25.8%) women overall reported that a midwife recommendation had been a reason for choosing influenza immunisation during pregnancy.

Pregnant women increasingly sought influenza vaccine. Whereas 8.8% (32/368) of vaccinated women recalled seeking vaccination in 2010 to 2013, the proportion of vaccinated women who remember asking for vaccination was much higher at 42% (41/98) in 2014.

Table 3 lists reasons cited by women for not being vaccinated during pregnancy. The two most common reasons did not change with time. Around one-half (50.9%) of non-vaccinated women did not recall influenza vaccination being mentioned by midwives or doctors and a similar proportion (47.1%) reported choosing against vaccination during pregnancy because they are not usually vaccinated when not pregnant.

**Table 3:** **Reasons women gave for electing AGAINST influenza immunisation in pregnancy** ^**a**^

	**Number of women stating not vaccinated during pregnancy**	**No one mentioned flu vaccination**		**GP advised against vaccination**		**Obstetrician advised against vaccination**		**Midwife advised against vaccination**		**Concerned about maternal vaccination effects**		**Concerned about baby vaccination effects**		**Not pregnant during the flu season**		**First trimester so avoided vaccine**	
**Year**	**N**	**n**	**n/N (%)**	**n**	**n/N (%)**	**n**	**n/N (%)**	**n**	**n/N (%)**	**n**	**n/N (%)**	**n**	**n/N (%)**	**n**	**n/N (%)**	**n**	**n/N (%)**
2010	139	75	54.0%	6	4.3%	1	0.7%	2	1.4%	64	46.4%	84	60.9%	0	0.0%	0	0.0%
2011	146	65	44.5%	6	4.1%	2	1.4%	0	0.0%	57	39.0%	62	42.5%	4	2.8%	1	0.7%
2012	131	72	55.0%	8	6.1%	3	2.3%	5	3.8%	32	24.4%	34	26.0%	1	0.8%	0	0.0%
2013	114	60	52.6%	2	1.8%	0	0.0%	0	0.0%	21	18.4%	25	21.9%	2	1.8%	0	0.0%
2014	91	44	48.4%	0	0.0%	0	0.0%	0	0.0%	27	29.7%	28	30.8%	1	1.1%	0	0.0%
	Chi-square, (df)	4.1, (4)		32.7, (3)		19.7, (3)		5.6, (3)		29.5, (4)		54.6, (4)		4.7, (4)		1.9, (2)	
	p	0.388				^b^		^b^		<0.001*		<0.001*		^b^		^b^	

*The Pearson Chi-square statistic is significant at the .01 level.

^a^Women could nominate more than one reason.

^b^Chi-square not calculated as > 20% of cells have expected cell counts < 5.

Maternal concerns about vaccine side effects became less common over time. The proportion of non-vaccinated women expressing concerns about safety for the fetus or infant almost halved from 60.9% in 2010 to 30.8% in 2014 (p < 0.001). The proportion of non-vaccinated women reporting concerns about vaccine side effects for themselves declined from 46.4% in 2010 to 29.7% in 2014 (p < 0.001). Overall, 35 of 1086 women (3.2%) reported receiving advice against influenza vaccination during pregnancy from a general practitioner, obstetrician or midwife (See Additional file 3). In 2013 to 2014 only 2 women (0.5%) reported this advice.

Regarding preferred sources of information about vaccination for 647 women interviewed in 2012 to 2014, 272 (42.0 %) of women nominated face to face discussion with a doctor or midwife. Slightly more women 345 (53.3%) were positive about receiving a text message reminder about influenza vaccination. Internet sourced information was also popular (125 women or 19.3%), more often mentioned than antenatal classes, brochures, email, books or mass media.

## Discussion

In the quest to protect pregnant women, their unborn and newly born children from influenza and its complications, our survey gives some cause for optimism. Vaccine coverage has increased from around 30% to 51% in the five years after 2009. Our findings are similar to those from a study conducted in the United States of America [12] as well as recent research from Western Australia [13]. It is uncertain the extent to which the processes of annual audit themselves promote vaccination, that is, a Hawthorne effect.

Infant or fetal benefits of influenza vaccination are increasingly common reasons for pregnant women to seek vaccination. Pregnant women’s decision-making for vaccination in favour of the fetus or newborn has been noted in quantitative [6] and qualitative [14] studies. We observed lower rates of concerns about vaccine side-effects for pregnant women and their babies in later compared with earlier surveys. Hospital consumer information and professional education about influenza vaccination addresses infant benefits and vaccine safety for mother and infant so our observations support the effectiveness of such education. We suggest that other antenatal vaccination programmes, such as pertussis, would be feasible with similar consumer information, professional education and logistic support.

Progressively fewer women nominated protection from severe maternal disease or recognised specific maternal risk factors as reasons for vaccination in the 5 years after the H1N1 pandemic. Close experience with a vaccine preventable disease is a recognised driver for immunisation [14]. Possibly memories of severe influenza illness among pregnant women during 2009 are fading. It is important for health care professionals and lay people to realise that pregnant women remain vulnerable to severe influenza disease in non-pandemic as well as pandemic years.

Although increasing, influenza vaccine coverage among childbearing women remains suboptimal. Health care provider advice is often the most important determinant of vaccine uptake [13]. Lack of discussion, rather than active advice against vaccination remains a common, potentially reversible reason for non-vaccination [12,13]. The observed rates of influenza vaccination uptake when a health care worker had recommended or discussed vaccination was 65%, very similar to comparable USA data of 70.5% [12]. This contrasts with 10.7% vaccination coverage among current study participants who did not recall a health care provider recommendation or discussion: very similar to the rate of 9.7% recorded in the comparable recent survey conducted in the USA [12]. Reports of HCW advising against influenza vaccination (in just over 3% of current survey participants) were not validated externally. These reports appear to be reducing over recent years. Participants, healthcare professionals or both may have confused recommendations about when to vaccinate against influenza with postpartum recommendations to vaccinate against diseases such as rubella and varicella zoster which require live vaccination. Participants and/or health care professionals may also have confused recommended antenatal influenza vaccination timing with pertussis postnatal timing (cocooning strategies) which were recommended at the time of survey.

Strengths of our study include good acceptability of the survey to newly postpartum women and thus high uptake which makes the findings generalizable to our tertiary hospital setting. Survey participants were representative of maternal and gestational age, and of cultural and linguistic diversity served by this hospital. Missing data from the 2012 survey could introduce unknown biases, but the response rate of close to 80%, compares favourably with other publications: more than half of surveillance reports of influenza vaccination during pregnancy published up to 2013 reported response rates of < 70% [6].

That newly postpartum women in the current study are well disposed towards text message reminders about vaccination during pregnancy encourages programmes similar to those used in an urban USA research setting which confirmed modest but positive increased uptake of influenza vaccination among pregnant women when serial text messages, linked to an electronic health record, are sent during autumn and winter [15].

Our study has some weaknesses. We relied on patient self-report of vaccination and the anonymised nature of the survey prevented confirmation. Nevertheless, other studies confirm accuracy of self-report [16,17]. Data are limited to a single institution rather than multi-centre [16,18]. Regardless, our findings support local vaccine promotion strategies within a national vaccination programme. Our study lacks information about education or income for respondents. Anecdotally, some women now expect influenza vaccination every pregnancy, particularly if they were pregnant during the 2009 H1N1 pandemic, but we did not have information about previous pregnancies so cannot examine this impression quantitatively. The national Woman Held Pregnancy Record [19] contains a field to document influenza vaccination during pregnancy thus supporting integration into routine pregnancy care.

## Conclusions

We surveyed new mothers as inpatients between 2010 and 2014 and were heartened to find influenza vaccine coverage during pregnancy is now more than 50%. There is still scope to protect more individual women and infants and increase herd immunity. Survey responses suggest that messages about vaccine safety in pregnancy and infant benefits are increasingly being heeded.

Survey responses indicate progressive lower awareness of maternal benefits of influenza vaccination, especially for women with risk factors for severe disease such as overweight and obesity, smoking, lung disease or diabetes. Those working to increase lay and professional awareness of the gravity and preventability of maternal influenza infection need to engage with and listen to childbearing women and other stakeholders, and be honest and open about uncertainty and risks of disease or vaccination programmes may not succeed [20].

An important, potentially reversible obstacle to influenza vaccination during pregnancy is lack of recommendation from health care providers. Around 1 in 2 non-vaccinated women state that lack of discussion with a maternity care provider was a reason for non-vaccination, an obstacle also identified in Australia and other countries [6,12,13]. There is scope to use text message and internet to support face to face discussion about influenza vaccination in pregnancy between women and maternity caregivers.

## Additional files

Additional file 1:
**Audit Tool. Paper Interview record completed by researcher/assistant.**
Additional file 2: Table S1. Socio-demographic characteristics. Additional file 3: Table S2. Reports of advice from Health Care Workers (HCW) to avoid influenza vaccination during pregnancy.
