# Supplementary material for: Increasing uptake of influenza vaccine by pregnant women post H1N1 pandemic: a longitudinal study in Melbourne, Australia, 2010 to 2014
Source: BMC Pregnancy Childbirth. 2015 Mar 5;15:53. doi: 10.1186/s12884-015-0486-3 (PMC4352234; doi:10.1186/s12884-015-0486-3)
Supplement: Additional file 1: — Audit Tool. Paper Interview record completed by researcher/assistant. [file 12884_2015_486_MOESM1_ESM.docx]

**Appendix A: MHW Influenza vaccination audit 2012**

Date: Audit information provided _______ Consent given □

(Researcher initial)

| **1. Age**  Less than 20 □ 30-34 □  20-24 □ 35-39 □  25-29 □ 40 and over □ |
| --- |
| **2. (a) Country of birth ________________________**  **2. (b) If Australia, Indigenous &/or Torres Strait Islander** Yes □ No □  **2. (c) Did the woman require an interpreter?** Yes □ No □ |
| **3. At what gestation did you give birth? (In completed weeks)**  **20-27 weeks** □ **28-31** □ **32-36** □ **37-41** □ **≥42** □ |
| **4. Primary antenatal care provider**  Hospital □  GP □  Private obstetrician □  Other □ **Please nominate ___________________________** |
| 5. At any time during your pregnancy, did a doctor, nurse, midwife or other health care worker offer you a flu vaccination or tell you to get one?  Yes □ No □ |
| **6. Health professional who offered the influenza vaccination**  Midwife □  GP □  Obstetrician □  Resident or registrar □  Other □**Please nominate ___________________________** |
| 7. Did you get an influenza vaccination during your pregnancy?  Yes □ No □ Not sure □  **If yes, which trimester?**  1^st^ □ 2^nd^ □ 3^rd^ □ Not sure □ |

| **If YES**  **8. What were your reasons for getting a flu vaccination during your pregnancy?**  For each item, circle Y (Yes) if it was a reason for you or circle N (No) if it was not a reason or did not apply to you (circle as many that apply)  YES NO   1. I normally get the flu vaccine Y N 2. I was worried about getting ‘swine flu’ or the flu Y N 3. I have a chronic medical condition e.g. asthma, obese Y N 4. My midwife recommended it Y N 5. My GP recommended it Y N 6. My obstetrician recommended it Y N 7. Friends/family/media information Y N 8. I wanted to protect my baby from getting the flu in his/her first few months Y N   i. Other Y N  Please tell us your reasons:  __________________________________________________________________________ |
| --- |
| **If NO**  9. What were your reasons for not getting a flu vaccination during your pregnancy?  For each item, circle Y (Yes) if it was a reason for you or circle N (No) if it was not a reason or did not apply to you (circle as many that apply)  YES NO  a. My doctor/midwife didn’t mention anything about a flu vaccination  during my pregnancy Y N  b. I was worried about side effects of the flu vaccination for me Y N  c. I was worried that the flu vaccination might harm my baby Y N  d. I wasn’t pregnant during the flu season (May - August) Y N  e. I was in my first trimester during the flu season (May - August) Y N  f. I don’t normally get a flu vaccination Y N  g. Other Y N  Please tell us your reasons: ___________________________________________________________________________ |
| 1. Do you remember reading a brochure about flu and pertussis vaccination in your hospital information pack (show participant an example) mailed to you after you had booked in for antenatal care?   Yes □ No □ Unsure □ |
| 1. Did you hear about influenza vaccination by a text message or SMS    1. No    2. Yes, from Mercy Hospital for Women    3. Yes, from my family doctor/GP    4. Yes, from other health professional    5. Yes, from friends or family    6. Yes, from another source: Please say who |
| 1. Would you like an SMS or text message reminder to discuss flu vaccination during a future pregnancy?   Yes □ No □ Unsure □   1. Are there any other ways of obtaining information about flu vaccination during pregnancy which you would recommend?   No □ Yes □ Internet:/ Books/ Antenatal classes/ Other. |
