# Supplementary material for: Increasing uptake of influenza vaccine by pregnant women post H1N1 pandemic: a longitudinal study in Melbourne, Australia, 2010 to 2014
Source: BMC Pregnancy Childbirth. 2015 Mar 5;15:53. doi: 10.1186/s12884-015-0486-3 (PMC4352234; doi:10.1186/s12884-015-0486-3)
Supplement: Additional file 2: Table S1. — Socio-demographic characteristics. [file 12884_2015_486_MOESM2_ESM.docx]

Supplementary table 1: Socio-demographic characteristics.

|  |  | 2010 |  | 2011 |  | 2012 |  | 2013 |  | 2014 |  | All years |  |
| --- | --- | --- | --- | --- | --- | --- | --- | --- | --- | --- | --- | --- | --- |
| Sample size |  | 199 |  | 240 |  | 203 |  | 252 |  | 192 |  | 1086 |  |
|  |  | N | (%) | N | (%) | N | (%) | N | (%) | N | (%) | N | (%) |
| Maternal age ^1^ | < 20 years | 3 | (1.5) | 2 | (0.8) | 2 | (1.0) | 3 | (1.2) | 3 | (1.6) | 13 | (1.2) |
|  | 20 to 24 years | 14 | (7.0) | 17 | (7.1) | 15 | (7.4) | 23 | (9.1) | 8 | (4.2) | 77 | (7.1) |
|  | 25 to 29 years | 44 | (22.1) | 62 | (25.8) | 56 | (27.6) | 69 | (27.4) | 47 | (24.6) | 278 | (25.6) |
|  | 30 to 34 years | 89 | (44.7) | 95 | (39.6) | 74 | (36.5) | 85 | (33.7) | 81 | (42.4) | 424 | (39.10 |
|  | 35 to 39 years | 39 | (19.6) | 47 | (19.6) | 45 | (22.2) | 54 | (21.4) | 42 | (22.0) | 227 | (20.9) |
|  | 40 to 44 years | 10 | (5.0) | 17 | (7.1) | 11 | (5.4) | 18 | (7.1) | 10 | (5.2) | 66 | (6.1) |
| Overseas-born ^2^ |  | 68 | (34.2) | 90 | (37.5) | 86 | (42.4) | 89 | (35.3) | 77 | (40.10 | 410 | (37.8) |
| Indigenous ^3^ |  | 0 | (0.0) | 3 | (1.3) | 2 | (1.0) | 3 | (1.2) | 1 | (0.5) | 9 | (0.8) |
| Private obstetric care |  | 28 | (14.1) | 26 | (10.8) | 24 | (11.8) | 23 | (9.1) | 13 | (6.8) | 114 | (10.5) |

^1^ 1 case of missing data, 2014, ^2^. 1 case of missing data, 2012 ^3^ 3 cases of missing data 2014

No trends were identified in maternal age structure or proportions of women who were overseas-born, identified as indigenous or used private obstetric care (chi-square test).
