# Supplementary material for: Increasing uptake of influenza vaccine by pregnant women post H1N1 pandemic: a longitudinal study in Melbourne, Australia, 2010 to 2014
Source: BMC Pregnancy Childbirth. 2015 Mar 5;15:53. doi: 10.1186/s12884-015-0486-3 (PMC4352234; doi:10.1186/s12884-015-0486-3)
Supplement: Additional file 3: Table S2. — Reports of advice from Health Care Workers (HCW) to avoid influenza vaccination during pregnancy. [file 12884_2015_486_MOESM3_ESM.docx]

Supplementary table 2:

Reports of advice from Health Care Workers (HCW) to avoid influenza vaccination during pregnancy

| Year | GP^1^ | Obstetrician | Midwife | Any HCW^2^ | Total | % |
| --- | --- | --- | --- | --- | --- | --- |
| 2010 | 6 | 1 | 2 | 9 | 199 | 4.5% |
| 2011 | 6 | 2 | 0 | 8 | 240 | 3.3% |
| 2012 | 8 | 3 | 5 | 16 | 203 | 7.9% |
| 2013 | 2 | 0 | 0 | 2 | 252 | 0.8% |
| 2014 | 0 | 0 | 0 | 0 | 192 | 0.0% |

^1^ General practitioner ^2^ HCW: health care worker
